# Glycolysis, monocarboxylate transport, and purinergic signaling are key events in *Eimeria bovis*-induced NETosis

**DOI:** 10.3389/fimmu.2022.842482

**Published:** 2022-08-11

**Authors:** Iván Conejeros, Sara López-Osorio, Ershun Zhou, Zahady D. Velásquez, María Cristina Del Río, Rafael Agustín Burgos, Pablo Alarcón, Jenny Jovana Chaparro-Gutiérrez, Carlos Hermosilla, Anja Taubert

**Affiliations:** ^1^ Institute of Parasitology, Justus -Liebig University Giessen, Giessen, Germany; ^2^ CIBAV Research Group, Facultad de Ciencias Agrarias, Universidad de Antioquia (UdeA), Medellín, Colombia; ^3^ College of Life Sciences and Engineering, University of Foshan, Foshan, China; ^4^ Department of Animal Pathology, Faculty of Veterinary Medicine, Universidad de Las Palmas de Gran Canaria, Las Palmas, Spain; ^5^ Laboratory of Inflammation Pharmacology, Faculty of Veterinary Science, Institute of Pharmacology and Morphophysiology, Universidad Austral de Chile, Valdivia, Chile

**Keywords:** *Eimeria bovis*, PMN, immunometabolism, NET formation, cattle, ECAR, autophagy

## Abstract

The protozoan parasite *Eimeria bovis* is the causative agent of bovine coccidiosis, an enteric disease of global importance that significantly affects cattle productivity. Previous studies showed that bovine NETosis—an important early host innate effector mechanism of polymorphonuclear neutrophil (PMN)—is elicited by *E. bovis* stages. So far, the metabolic requirements of *E. bovis*-triggered NET formation are unknown. We here studied early glycolytic and mitochondrial responses of PMN as well as the role of pH, distinct metabolic pathways, P2 receptor-mediated purinergic signaling, and monocarboxylate transporters 1 and 2 (MCT1, MCT2) in *E. bovis* sporozoite-induced NET formation. Seahorse-based experiments revealed a rapid induction of both neutrophil oxygen consumption rate (OCR) and early glycolytic responses, thereby reflecting immediate PMN activation and metabolic changes upon confrontation with sporozoites. The impact of these metabolic changes on NET formation was studied *via* chemical inhibition experiments targeting glycolysis and energy generation by the use of 2-fluor-2-deoxy-D-glucose (FDG), 6-diazo-5-oxo-L-norleucin (DON), sodium dichloroacetate (DCA), oxythiamine (OT), sodium oxamate (OXA), and oligomycin A (OmA) to block glycolysis, glutaminolysis, pyruvate dehydrogenase kinase, pyruvate dehydrogenase, lactate dehydrogenase, and mitochondrial ATP-synthase, respectively. Overall, sporozoite-induced NET formation was significantly diminished *via* PMN pretreatments with OmA and OXA, thereby indicating a key role of ATP- and lactate-mediated metabolic pathways. Consequently, we additionally studied the effects of extracellular pH, MCT1, MCT2, and purinergic receptor inhibitors (AR-C141900, AR-C155858, theobromine, and NF449, respectively). Pretreatment with the latter inhibitors led to blockage of sporozoite-triggered DNA release from exposed bovine PMN. This report provides first evidence on the pivotal role of carbohydrate-related metabolic pathways and purinergic receptors being involved in *E. bovis* sporozoite-induced NETosis.

## Introduction

*Eimeria bovis* is an obligate intracellular apicomplexan parasite of cattle, which may cause severe hemorrhagic typhlocolitis in calves thereby producing high economic losses worldwide in cattle industry (1). Even though early host innate defense reactions should be critical for the outcome of the disease, few studies have been performed on early host innate immune reactions being directed against *E. bovis* stages (2–6). This evidence indicates that polymorphonuclear neutrophils (PMN) play an important role in early immune responses against this parasite. For example, PMN infiltrate parasitized tissue and accumulate at the site of meront formation. In addition, since *E. bovis* sporozoites must traverse the mucosal layer to reach the lymphatic endothelial cells (4, 6, 7), the interaction of *E. bovis* sporozoites with PMN results in enhanced production of pro-inflammatory cytokines, chemokines, oxidative burst/iNOS activities, and neutrophil extracellular trap (NET) formation, also known as NETosis. Furthermore, neutrophil phagocytic and oxidative burst activities increase in response to *E. bovis* sporozoites *in vitro* or *ex vivo* during infection (4). Notably, *E. bovis* represent potent inducers of NETs in the bovine system not only *in vitro* but also *in vivo* (8). PMN-derived NET-like structures were demonstrated to firmly attach to these parasitic stages, decreasing the host cell invasion (7). Muñoz-Caro et al. (6) explored in more depth molecular mechanisms of *E. bovis*-induced NETosis and found that it depended on NADPH oxidase (NOX), neutrophil elastase (NE), and myeloperoxidase (MPO) activities, on reactive oxygen species (ROS) generation and intracellular calcium mobilization from store-operated calcium entry (SOCE) sources. Furthermore, PMN-derived CD11b is involved in NETosis since antibody-mediated blockage of this receptor led to an effective diminishment of NET release (6). As also described for other inducers of NETosis (9, 10), *E. bovis* sporozoite-mediated NET formation revealed to be regulated by ERK1/2 and p38 MAPK-dependent pathways (6). In addition, sporozoites, merozoites, and oocyst stages of *E. bovis* were also proven as NETosis triggers, indicating that this process is not stage-specific (6).

Autophagy is a lysosomal degradation pathway that is essential for survival, differentiation, development, and homeostasis (11) and is also present in PMN (12). First evidence suggests that autophagy is necessary for and related to NET formation. In the case of *Besnoitia besnoiti*, a closely related apicomplexan parasite that also affects cattle, NETosis occurred in parallel with increased autophagic activities in tachyzoite-exposed bovine PMN (13). Besides other molecules, autophagy is regulated by the metabolic sensor AMP-activated kinase α (AMPKα) and by the mechanistic target of rapamycin (mTOR) (14). Autophagy was shown as crucial in regulating early innate effector mechanisms against pathogens, such as phagocytosis (15) and NET formation (12, 16, 17). Nevertheless, the precise role of autophagy in parasite-triggered NET formation is still a matter of study.

Two main mechanisms of NET release were described so far, “suicidal” and “vital” NETosis. NET formation linked to cell death is a slow process (120–240 min) and depends on the classic pathways of the NETotic cell death process, such as NOX activities and ROS production. This mechanism is also known as suicidal NETosis (9, 18). In addition, an alternative, rapid process (5–60 min) of NET formation was reported in which PMN still perform some phagocytic function and chemotaxis. This type is called vital NETosis (18–20) and is described as ROS-independent in response to some pathogens (21, 22). Additionally, vital NETosis includes vesicular DNA movement from the nucleus to the extracellular space (19). Within this pathway, the membrane integrity is maintained, and it does not require the death of PMN (23). Currently, there is no information on the presence of these NETosis types in sporozoite-exposed bovine PMN.

So far, data on metabolic requirements of parasite-induced NETosis are scarce even though a positive correlation between cell activation and metabolic pathway induction is well-documented in various innate immune cells. Thus, immune cells are described to shift between resting and activated states by switching energy-producing pathways on and off (24, 25). Recent analyses have shown that PMA stimulation led to enhanced glucose consumption and glycolytic rates in human PMN (26) and PMA-induced NETosis was confirmed as glycolysis-dependent since glycolysis inhibition effectively blocked NET formation (26, 27). In line with this, high-glucose *in vitro* conditions mediated enhanced NETosis rates which were also found under hyperglycemia and type 2 diabetes (28). On the other hand, *B. besnoiti*-induced DNA release, measured as cell-free NETs, depends on mitochondrial activities but not on glycolysis (29).

Monocarboxylate transporters (MCT) catalyze the bidirectional proton-linked transport of short-chain monocarboxylates, such as L-lactate and pyruvate, across the plasma membrane of mammalian cells (30) and are therefore essential for pH homoeostasis. D-Lactate induces NET release in cattle *via* monocarboxylate transporter 1 (MCT1) (31). Furthermore, MCT—among other fatty and hydroxycarboxylic acid receptors—were proposed as critical modulators of cattle innate immune responses in common metabolic disorders such as laminitis and ruminitis (32). Lactate is also actively released from activated PMN (33), and glycolysis-derived lactate is linked to NET production in human PMN (34). Successful NET formation depends on ATP-based energy supply generated *via* glycolysis. This ATP is required for active cytoskeletal rearrangements preceding NET extrusion (35). Of further note, mitochondrial ATP synthesis mediates mitochondrial hyperpolarization and NET formation induced by the platelet-activating factor (PAF) in bovine PMN (36). Interestingly, extracellular purines are shown to differentially influence innate immune cell effector functions. While extracellular adenosine inhibited PMA-triggered NETosis (37), extracellular ATP generally upregulates effector functions in PMN, macrophages, or dendritic cells by triggering pro-inflammatory cytokine production, inflammasome assembly, and migration (38). In bovines, the fast oleic and linoleic acid-driven ATP release in PMN also regulates the release of NET-associated DNA (39). Likewise, extracellular ATP disposal and activation of P2-receptor-mediated purinergic signaling pathways seem essential for early host innate immune responses of PMN (40, 41). P2-receptor-mediated purinergic signaling was also involved in other essential PMN functions, such as chemotaxis, phagocytosis, oxidative burst, and degranulation (40), and has been recently proven as essential for *Neospora caninum*-driven NET formation since tachyzoite-mediated NETosis was significantly blocked by a P2Y2 receptor inhibitor (42). Also, treatments with the purinergic inhibitor of P2X1 NF449 significantly decreased NET formation induced by *Besnoitia besnoiti* tachyzoites (29) or *Trypanosoma brucei brucei* trypomastigotes (43).

The current work aimed to analyze in more detail the metabolic requirements of *E. bovis* sporozoite-triggered NETosis. Therefore, we explored bovine PMN-derived glycolytic responses and oxygen consumption rate (OCR) after sporozoite exposure and studied the role of distinct metabolic pathways, purinergic receptors, and MCTs in *E. bovis*-triggered NETosis *via* functional inhibition assays. Additionally, we analyzed autophagosome formation in the dynamic parasite-mediated NETosis process and generated the first evidence of *E. bovis-*stimulated PMN undergoing vital NETosis by using a novel live-cell 3D-holotomographic microscopy.

## Materials and methods

### Ethics statement

Animal trials were conducted following the Justus Liebig University (JLU) Giessen Animal Care Committee Guidelines. Protocols were approved by the Ethics Commission for Experimental Animal Studies of Federal State of Hesse (Regierungspräsidium Giessen; A2/2016; JLU-No. 589_AZ and G16/2017, JLU-No. 835_GP) and in accordance with European Animal Welfare Legislation: ART13TFEU and current applicable German Animal Protection Laws.

### Parasites

*Eimeria bovis* strain H was originally isolated in the field in northern Germany in 1985 and maintained by passages in calves (3). For oocyst production, two 8-week-old calves, kept in *Eimeria*-free housing conditions in special metabolic cages, were infected orally with 3 × 10^4^ sporulated *E. bovis* oocysts. Oocysts were isolated from feces beginning at 18–20 days *post infectionem* (p.i.) according to Jackson (44). The feces were washed with tap water through a set of sieves (pore sizes 850, 250, and 80 µm), and the resulting suspension was sedimented overnight. This sediment was mixed at a 1:1 ratio with a sucrose-saturated solution (ρ = 1.3 g/ml) and adjusted to a final density of 1.15 g/ml. For oocyst flotation, the fecal suspension was carefully transferred into plastic trays (30 × 20 × 5 cm). The trays were filled up and covered with clean glass plates, guaranteeing contact of the suspension with the surface of the glasses. Every 4 h, the glass plates were carefully removed, and adherent oocysts were collected by washings with tap water. The remaining fecal suspension was mixed, filled up, and subjected to further oocyst flotation. The process was repeated until a few oocysts were left in the suspension (microscopic control: less than 5 oocysts per power vision field of ×10 magnification). Oocyst suspension was diluted with water (1:1) and centrifuged (600 × *g*, 12 min). An oocyst pellet was suspended in potassium dichromate solution (2% *w/v* final concentration, Merck) and incubated at room temperature (RT) with constant aeration for adequate oocyst sporulation. When ≥90% of the oocysts achieved sporulation, the oocysts were pelleted (600 × *g*, 12 min), suspended in fresh 2% (*w/v*) potassium dichromate solution, and stored at 4°C until further use (5).

For sporozoite isolation, the protocol described by Silva et al. (45) was used: sporulated oocysts were incubated in 4% (*v/v*) sodium hypochlorite solution and stirred on ice for 20 min. After vortexing for 20 s, the oocyst solution was centrifuged (300 × *g*, 5 min) and the supernatant was mixed with bi-distilled water (1:1). This suspension was then filtered through sieves of 40- and 10-µm pore size (pluriStrainer, pluriSelect) to remove debris. Then, oocysts were pelleted (15 min, 600 × *g*), suspended in 0.02 M L-cysteine/0.2 M NaHCO_3_ (Merck) solution, and incubated at 100% CO_2_ atmosphere (37°C, 20 h). Thereafter, oocysts were pelleted (15 min, 600 × *g*) and incubated (up to 3 h, 37°C, 5% CO_2_ atmosphere) in the following excystation medium: Hank’s balanced salt solution (HBSS, Gibco) containing 0.4% (*w/v*) trypsin (Sigma) and 8% (*v/v*) sterile-filtered bovine bile obtained from a local slaughterhouse. The excystation progress was microscopically controlled every hour. Freshly released sporozoites were collected, filtered through a 5-µm filter unit (pluriSelect), washed twice (600 × *g*, 15 min) with cell culture medium 199 [M199, Gibco, supplemented with 2% (*v/v*) fetal calf serum (FCS, Gibco), 1% penicillin (*v/v*, 500 U/ml; Sigma-Aldrich), and streptomycin (*v/v*, 500 μg/ml; PS; Sigma-Aldrich)], and finally suspended in RPMI 1640 medium (Sigma-Aldrich) at 2 × 10^6^ sporozoites/ml.

### Isolation of bovine PMN

Healthy adult dairy cows (*n* = 9) served as blood donors. Animals were bled by puncture of the jugular vein, and 30 ml peripheral blood was collected in heparinized sterile plastic tubes (Kabe Labortechnik). Twenty milliliters of heparinized blood was diluted in 20 ml sterile PBS with 0.02% EDTA (Sigma-Aldrich), layered on top of 12 ml Biocoll^®^ separating solution (density = 1.077 g/l; Biochrom AG), and centrifuged at 800 × *g* for 45 min. After removal of plasma and peripheral blood mononuclear cells (PBMCs), the cell pellet was suspended in 27 ml bi-distilled water and gently mixed for 30 s to lyse erythrocytes. Osmolarity was rapidly restored by adding 3 ml of 10× Hank’s balanced salt solution (HBSS; Biochrom AG). For full erythrocyte lysis, this step was repeated twice and PMN were suspended in sterile RPMI 1640 medium (Sigma-Aldrich). PMN were counted in a Neubauer hemocytometer. Finally, freshly isolated bovine PMN were allowed to rest (37°C, 5% CO_2_ atmosphere) for 30 min until further use.

For Seahorse XF-based experiments, the protocol of erythrocyte lysis was slightly modified. In brief, plasma and buffy coat containing PBMCs were aspirated following Biocoll^®^ gradient centrifugation. The remaining cell pellet was suspended in Hank’s balanced salt solution (HBSS; Biochrom AG). Red blood cells were removed by flash hypotonic lysis using one volume of an ice-cold phosphate-buffered water solution (5.5 mM NaH_2_PO_4_, 8.4 mM HK_2_PO_4_, pH 7.2). After 1 min of incubation, two volumes of ice-cold hypertonic phosphate-buffered solution (5.5 mM NaH_2_PO_4_, 8.4 mM HK_2_PO_4_, 0.46 M NaCl, pH 7.2) were supplemented to restore isotonicity. Then, the cells were pelleted (600 × *g*, 10 min) and suspended in 5 ml of HBSS. This lysis step was repeated until no erythrocytes were observed in the cell preparation as described in (46, 47). PMN were then centrifuged at 600 × *g* for 10 min and washed with HBSS, counted, and processed as described above. The purity of the PMN suspension was assessed in an automatic cell counter Olympus R1 (Olympus). Values of cell purity were always >92% independent of the PMN isolation protocol.

### Bioenergetic PMN analysis

#### Estimation of oxygen consumption rate and extracellular acidification rate in bovine PMN

In total, 1 × 10^5^ PMN (*n* = 3) were seeded in duplicates into poly-_L_-lysine (0.001%)-coated XFp plates (Agilent) using XF RPMI medium as vehicle (XF RPMI supplemented with 2 mM L-glutamine, 1 mM pyruvate, and 10 mM glucose, final concentrations; Agilent). Oxygen consumption rate (OCR) and extracellular acidification rate (ECAR) in bovine PMN were determined by a Seahorse XF Analyzer (Agilent) according to the manufacturer’s instructions. This system allows non-invasive and real-time measurements of OCR and proton efflux rates (PER, mirroring total extracellular acidification), which correlate with mitochondrial function, oxidative burst, and glycolytic activities (48). Changes in PMN-derived OCR and ECAR/PER were measured in response to *E. bovis* sporozoite (1:1) exposure in the presence of the mitochondrial inhibitors rotenone (Rot) and antimycin A (AA) (0.5 µM final concentration). The supplementation of mitochondrial inhibitors *via* injection ports of the instrument was performed to block oxygen consumption due to mitochondrial respiration. Therefore, sporozoites were added to PMN *via* an internal injection port of the instrument after 30 min of basal neutrophil-derived OCR/ECAR estimation.

#### Estimation of glycolytic responses in bovine PMN

To address the glycolytic function of *E. bovis*-exposed PMN, we used Agilent Seahorse XF Glycolysis Stress Test (Kit 103017-100). PMN (*n* = 3, 1 × 10^5^ cells) were seeded in duplicates into poly-L-lysine (0.001%)-coated XFp plates (Agilent) in XF assay media (XF RPMI supplemented with 2 mM L-glutamine, 1 mM pyruvate, and 10 mM glucose, final concentrations; Agilent). Basal PMN metabolism (ECAR [mpH/min] and OCR [pmol/min]) was measured for 15 min to define a baseline. Thereafter, *E. bovis* sporozoites (1 × 10^5^) were injected *via* an internal port of the instrument and basal parameters were measured for another 15 min in sporozoite-confronted bovine PMN. Afterwards, ECAR and OCR were measured applying consecutive injections of glucose (10 mM; glycolysis substrate), oligomycin (1 µM; inhibits ATP synthase), and 2‐DG (50 mM; a competitive inhibitor of glycolysis) following the manufacturer’s instructions. The kit-based analysis provides the following carbohydrate metabolism parameters: glycolysis, glycolytic reserve, glycolytic capacity, and non-glycolytic acidification, which were all calculated following the manufacturer’s instructions using Wave software (Agilent). To calculate basal glycolysis, the software uses the average of the three measurements after glucose injection (subtracting non‐glycolytic acidification). Glycolytic capacity was calculated using three measurements after oligomycin injection and further non‐glycolytic acidification subtraction. From the registries, then, the glycolytic reserve and the total glycolytic capacity were calculated. Finally, non‐glycolytic acidification corresponds to an average of the first and last three measurements of ECAR values.

To assess the effectivity of 2-fluor-2-deoxy-D-glucose (FDG) 2 mM in bovine PMN, we used Agilent Seahorse XF^®^ Glycolysis Stress Test (Kit 103017-100) with PMN. PMN (1 × 10^5^, *n* = 3) were seeded in duplicates into poly-L-lysine 0.001%-coated XFp plates (Agilent) in XF assay media for use with the glycolysis stress kit (Seahorse Bioscience). Basal ECAR and OCR were measured in PMN for 15 min and then FDG (2 mM) was injected *via* an internal port of the instrument. After 15 min of injection, the glycolytic response of PMN was measured (ECAR [mpH/min]) as described above.

### Autophagosome detection by immunofluorescence analysis

Analysis of autophagosome formation in PMN was performed according to Itakura and McCarty (49). Bovine PMN (*n* = 3) were deposited on poly-_L_-lysine (0.01%)-pretreated coverslips (15-mm diameter, Thermo Fisher Scientific). In addition, pretreatments of PMN with rapamycin (50 nM) or wortmannin (50 nM) were performed for 30 min before exposure to *E. bovis* sporozoites (1:4 PMN:sporozoite ratio, 2 h). After incubation, cells were fixed with 4% paraformaldehyde (10 min; Merck), permeabilized with ice-cold methanol (Sigma-Aldrich) treatment (3 min at 4°C), and blocked with blocking buffer (5% BSA, 0.1% Triton X-100 in sterile PBS; all Sigma-Aldrich) for 60 min at RT. Thereafter, cells were incubated overnight at 4°C in anti-LC3B antibody solution (Cat# 2775 Cell Signaling Technology) diluted 1:200 in blocking buffer. After incubation, samples were washed three times with PBS and incubated for 30 min in the dark at RT in a 1:500 dilution of goat anti-rabbit IgG conjugated with Alexa Fluor 488 (Invitrogen). After three washes in sterile PBS, samples were mounted in ProLong anti-fading mounting media containing DAPI (Invitrogen) on glass slides and images were taken applying confocal microscopy (Zeiss LSM 710). To estimate LC3B-positive cells, the background fluorescence signal was determined in control conditions for FITC (green) and DAPI (blue) channels and the green fluorescence intensity was determined. An LC3B-positive cell was defined using two parameters: green fluorescence and the presence of LC3B puncta as described by Itakura and McCarthy (49). Image processing was carried out with Fiji ImageJ using Z-project and merged channel plugins and restricted to overall adjustments of brightness and contrast.

### Detection and image-based quantification of *E. bovis*-triggered NETs

For NET analyses, 2.5 × 10^5^ PMN from three different donors (*n* = 3) were seeded on 12-mm glass coverslips (Thermo Fisher) coated with 0.01% poly-_L_-lysin (Sigma) and left unstimulated for 30 min at 37°C and 5% CO_2_. Then PMN were confronted with freshly excysted *E. bovis* sporozoites at 1:2 and 1:4 PMN/sporozoite ratios in HBSS for 2 h at 37°C and 5% CO_2_. After incubation, the samples were fixed with paraformaldehyde (Merck) at 4% final concentration for 15 min at RT, carefully washed thrice with sterile PBS, and stored at 4°C until immunostaining was performed. Immunodetection of NETs was performed by use of anti-histone-DNA complex antibodies (Millipore Cat#MAB3864; 1:100 dilution, detected with Invitrogen’s AlexaFluor 647 Cat#A21235 1:500) and anti-neutrophil elastase (anti-NE; Abcam, Cat#Ab68672 1:200 dilution; detected with Invitrogen’s AlexaFluor 594 Cat#R37117 1:500 dilution) and counterstained for DNA using DAPI in the mounting medium (Fluoromount-G with DAPI, Dako). All antibodies were diluted in permeabilization/blocking solution consisting of PBS with 3% BSA and 0.3% Triton X-100 (all Sigma-Aldrich). The incubation period was overnight at 4°C for primary antibodies. Thereafter, samples were washed thrice with PBS. Incubation with secondary antibodies was performed for 30 min at RT, and the coverslips were washed thrice with PBS and mounted on the microscope slide. The samples were kept protected from light for 24 h at RT before analysis. Imaging was performed by a Nikon Eclipse Ti2-A inverted microscope equipped with ReScan confocal microscopic instrumentation (RCM 1.1 Visible, Confocal.nl) and a motorized Z-stage (DI1500). Image acquisition was performed using the NIS-Elements v 5.11 software (Nikon) applying a z-stack optical series with a step size of 0.1 microns. Z-series were displayed as maximum z-projections maintaining the brightness and contrast conditions within the datasets of each biological experiment using ImageJ Fiji version (50).

The percentage of cells releasing NETs was assessed as described by Brinkmann et al. (51) with minor modifications. Three random images per animal (*n* = 3) were obtained at ×10 magnification using a confocal microscope. A manual threshold was applied to each channel using the clustering algorithm of Otsu, and the total number of particles was counted.

### Live-cell 3D holotomographic microscopy of NET-forming bovine PMN

To illustrate early interactions of PMN with vital and motile *E. bovis* sporozoites, live-cell 3D holotomographic videos were recorded. In total, 5 × 10^5^ PMN were seeded into a 35-mm tissue culture µ-dish (ibidi) in imaging medium [RPMI 1640 lacking phenol red and serum, supplemented with 0.5 µM SYTOX Green (Life Technologies) and 2 µM DRAQ 5 (Thermo Scientific)]. After 30 min of incubation in the ibidi^®^ Stage Top Incubation Systems, *E. bovis* sporozoites were added (1:1, 5 × 10^5^). Then, holotomographic videos were obtained by using a 3D Cell Explorer microscope (Nanolive 3D) equipped with a ×60 magnification (λ = 520 nm, sample exposure 0.2 mW/mm^2^) and a depth of field of 30 µm. The FITC channel was used to visualize extracellular DNA (present in NETs and death cells, = green fluorescence), and the TRITC channel (red fluorescence) was used for nuclear DNA visualization. The video was post-processed and analyzed using STEVE software (Nanolive).

### Quantification of ‘anchored’ and ‘cell free’ NETs in inhibition and pH-related experiments

Bovine PMN from three donors were suspended in RPMI 1640 medium lacking phenol red (Sigma) and serum. Then the PMN were confronted with viable *E. bovis* sporozoites at a final PMN: sporozoite ratio of 1:4 (2 h, 37°C, 5% CO_2_, 96-well plates). For negative controls, PMN or sporozoites in plain medium were used.

For pH-related experiments, RPMI 1640 medium was adjusted to different pH values (pH 6.6, 7.0, 7.4, and 7.8) by HCl or NaOH (both Merck, Darmstadt, Germany) supplementation as previously described (52). Bovine PMN were suspended in pH-adjusted RPMI 1640 medium and then exposed to sporozoites. Experiments were performed as follows: 2 × 10^5^ PMN were seeded in duplicates into 96-well plates and cocultured with 8 × 10^5^ *E. bovis* sporozoites or incubated in plain pH-adjusted medium (controls) for 2 h at 37°C and 5% CO_2_.

For inhibition assays, bovine PMN (*n* = 3) were pretreated with inhibitors for 30 min and then cocultured with *E. bovis* sporozoites (1:4 PMN: sporozoite ratio, 2 h, 37°C, 5% CO_2_). The following chemical blockers were used: 2-fluor-2-deoxy-D-glucose (FDG, 2 mM, Sigma-Aldrich; glucose analogue, inhibitor of glycolysis), sodium dichloroacetate (DCA, 8 mM, Sigma-Aldrich; inhibitor of pyruvate dehydrogenase kinase), oxythiamine (OT, 50 µM, Sigma-Aldrich; inhibitor of pyruvate dehydrogenase, α-ketoglutarate dehydrogenase, and transketolase), sodium oxamate (OXA, 50 mM, Sigma-Aldrich; structural analogue of pyruvate, inhibitor of lactate dehydrogenase), 6-diazo-5-oxo-L-norleucin (DON, 4 µM, inhibitor of glutaminolysis), oligomycin A (5 µM, Sigma-Aldrich; inhibitor of ATP-synthase in mitochondrial respiration), theobromine (100 µM, Sigma-Aldrich; inhibitor of P1A1-mediated purinergic signaling), NF449 (100 µM, Tocris; purinergic receptor antagonist with high specificity for P2X_1_), AR-C141990 (1 µM, Tocris; MCT1 inhibitor), and AR-C155858 (1 µM, Tocris; inhibitor of MCT1 and MCT2). Inhibitor concentrations were chosen according to previous studies (26, 42, 53–56). Stimulation of PMN with zymosan (1 mg/ml; Sigma-Aldrich) served as positive control.

For pH-related and inhibitor assay, ‘anchored’ and ‘cell-free’ NETs were distinguished according to Tanaka et al. (57). Therefore, the samples were directly centrifuged in the cell culture plate (300 × *g*, 5 min) after incubation. Supernatants were transferred to a new 96-well plate to measure ‘cell-free’ NETs, and the remaining pellets were used for ‘anchored’-NET estimation. For both sample types, 50 µl PicoGreen (Invitrogen, diluted 1:200 in 10 mM Tris base buffered with 1 mM EDTA) was added to each well. Extracellular DNA was quantified based on PicoGreen-derived fluorescence intensities using an automated multiplate reader (Varioskan, Thermo Scientific) at 484 nm excitation/520 nm emission (42, 58).

### Statistical analysis

Unless otherwise stated, comparisons between two groups were performed using a paired t-test analysis. When more than two datasets were analyzed, a paired one-way analysis of variance (ANOVA) followed by Dunnett’s multiple-comparison test was applied. Statistical significance was defined by a *p*-value < 0.05. All graphs (mean ± SEM) and statistical analyses were generated by GraphPad software (v. 7.03).

## Results

### *E. bovis* sporozoite exposure triggered oxygen consumption and glycolysis in bovine PMN

**Figure 1:**
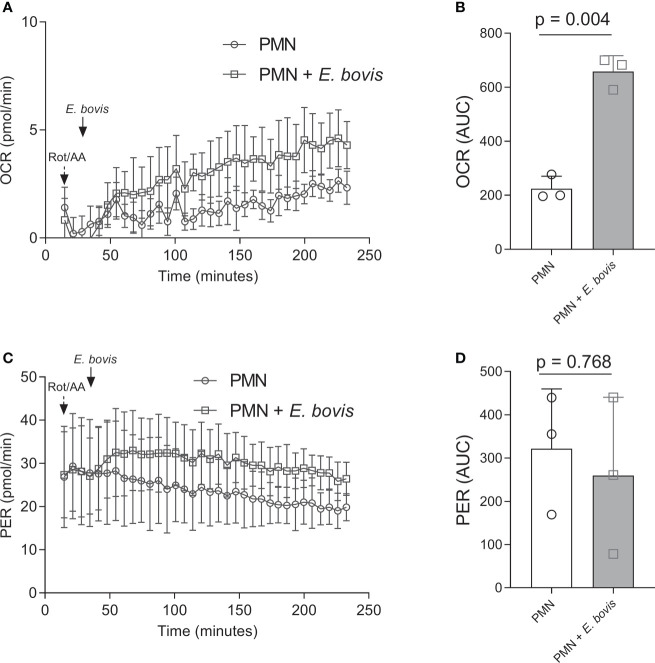
Oxygen consumption and extracellular acidification rates in *E. bovis* sporozoite-exposed bovine PMN. Oxygen consumption rate (OCR) and extracellular acidification rate (ECAR) expressed as proton efflux rate (PER) of bovine PMNs exposed to *E. bovis* sporozoites were measured by an extracellular flux analyzer (Seahorse Bioscience). A total of 100,000 PMNs were plated in eight-well XFp Agilent plates, and rotenone/antimycin A and *E. bovis* sporozoites were injected sequentially. OCR **(A)** and PER **(C)** were measured during 240 min, and the corresponding area under the curve of each registry **(B, D)** was calculated for each experimental condition to quantify the activation of bovine PMN. All data are shown as mean ± SEM; p values were calculated by a paired two-tailed t-test analysis (n = 3).

As a direct indicator of PMN activation, we here measured metabolic key functions in bovine PMN being exposed to *E. bovis* sporozoites. Thus, OCR were estimated *via* Seahorse-based technology over a time period of 4 h. After detecting basal respiration in non-exposed PMN for 30 min, sporozoites were added to resting PMN in the presence of the mitochondrial activity inhibitor rotenone/antimycin A. An immediate and ongoing increase in OCR was here detected in response to sporozoite exposure (**Figure 1A**). When analyzing the area under the curve (AUC), a significant overall induction of OCR was found (sporozoite-exposed PMN vs. non-exposed PMN: *p =* 0.004, **Figure 1B**), thereby reflecting PMN activation. Additionally, proton efflux rates (PER), reflecting ECAR, were measured in sporozoite-exposed PMN *via* Seahorse-based analyses. In contrast to OCR, no significant differences were here detected (*p* = 0.768) even though PER seemed moderately but constantly induced by sporozoite supplementation (**Figures 1C, D**).

**Figure 2:**
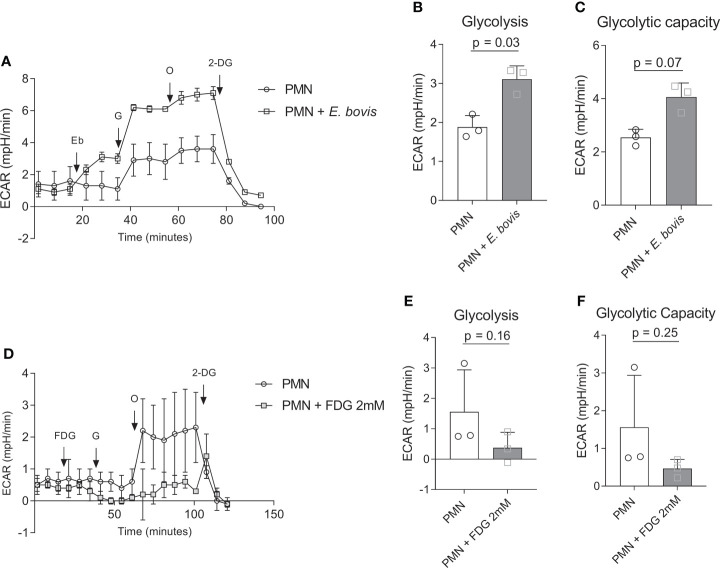
Glycolytic responses in *E. bovis* sporozoites-exposed bovine PMN. **(A)** Bovine PMN were resuspended in XF assay medium for glycolysis stress kit (Seahorse Bioscience). A total of 100,000 PMN were plated in eight-well XFp Agilent plates, and glycolytic responses were assessed as extracellular acidification rate (ECAR) after sequential injections of sporozoites (400,000), glucose (10 mM), oligomycin (1 µM; inhibits mitochondrial ATP synthase), and 2‐DG (50 mM, blocks glycolysis). Arrows indicate injections of *E. bovis* sporozoites (Eb), glucose (G), oligomycin (O), and 2-deoxyglucose (2-DG) **(B, C)**. **(D)** Glycolytic response of FDG-treated bovine PMN. Extracellular acidification rate (ECAR) reflecting glycolytic responses were measured after serial injections (indicated by arrows) of FDG (2 mM, blocks glycolysis), glucose (activates glycolysis, 10 mM), oligomycin (0.1 μM, inhibits mitochondrial ATP synthase), and 2-deoxy-D-glucose (DG, 50 mM, blocks glycolysis); **(E, F)** Glycolytic parameters in FDG-treated and non-treated bovine PMN. All data are presented as mean ± SEM. p values were calculated by a paired two-tailed t-test analysis (n = 3).

We furthermore analyzed whether sporozoite exposure would influence the glycolytic activity of bovine PMN. In the current Seahorse-based experimental setting (glycolysis stress test, without mitochondrial inhibitors), a short period (18 min) of basal ECAR estimation in non-exposed PMN was followed by sporozoite supplementation to bovine PMN (**Figure 2A**, indicated by an arrow + *Eb*). PMN confrontation with sporozoites led to an immediate and significant increase of ECAR, thereby reflecting an enhancement of the acute glycolytic response in exposed cells (**Figure 2A**). Additional glucose supplementation revealed that also the general glycolysis was significantly enhanced in PMN:sporozoite cocultures (sporozoite-exposed PMN vs. non-exposed PMN: *p* = 0.03) (**Figure 2B**). Also, ECAR measurements after the injection of oligomycin (blocks mitochondrial ATP production) revealed that glycolytic capacity was increased in PMN:sporozoite cocultures when compared to non-stimulated PMN. This increase did not reach statistical significance (sporozoite-exposed PMN vs. non-exposed PMN: *p* = 0.07) (**Figure 2C**). The contribution of *E. bovis*-sporozoites alone to these results is not significant given the low OCR and ECAR values measured in E. bovis-sporozoites using the same experimental settings (**Supplementary Figure 1**). Additionally, we performed glycolysis stress tests in the presence of 2 mM FDG, an analogue of glucose and inhibitor of glycolysis that cannot be metabolized. Results showed that FDG treatments indeed inhibited glycolysis, but this result was not statistically significant (*p* = 0.16). The same was observed when the glycolytic capacity of *E. bovis*-confronted PMN was evaluated (**Figures 2D–F**).

### *E. bovis* sporozoite exposure-induced autophagy in bovine PMN

**Figure 3:**
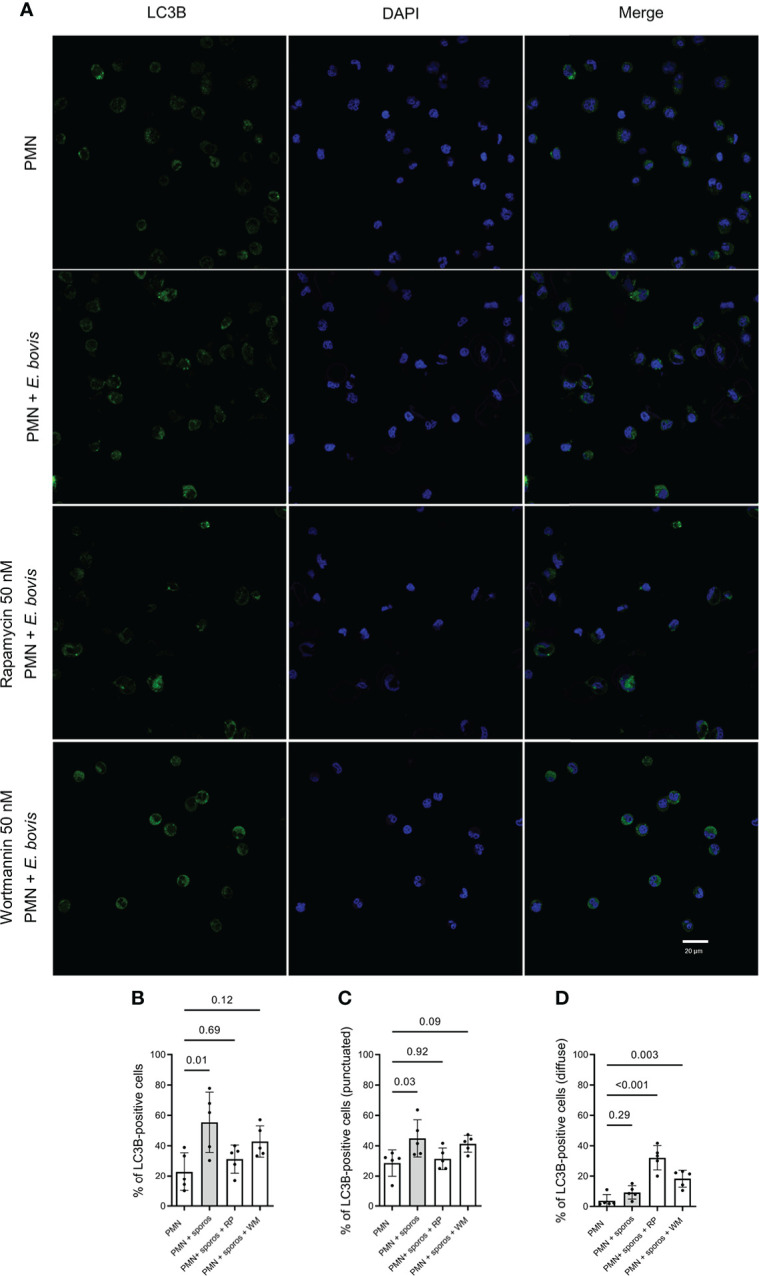
LC3B-based autophagosome formation in bovine *E. bovis* sporozoite-exposed PMN. Panel showing the staining for LC3B (green), DAPI (blue) phase-contrast (grayscale), and merge **(A)**. Also, PMN were pretreated with rapamycin (RP) or wortmannin (WT) (both 50 nM for 30 min) and then exposed to *E. bovis* sporozoites or plain medium (PMN). After 2 h of incubation, the samples were stained for LC3B and DNA and the number of autophagosome-positive cells was determined **(B)**. Also, the punctuated **(C)** or diffuse **(D)** signal of LC3B was analyzed. p-values were calculated using a repeated measures (paired) ANOVA followed by a Dunnett’s multiple-comparison test analysis (n = 5).

LC3B was detected in immunofluorescence analyses to evaluate the activation of autophagy in bovine PMN being confronted with *E. bovis* sporozoites. Here, non-stimulated PMN showed a non-punctuated, homogeneous distribution of the LC3B (green) signal (**Figure 3A**, first row). This pattern changed to a typically punctuated phenotype by the formation of autophagic vesicles upon sporozoite confrontation (**Figure 3A**, second row). Furthermore, we performed co-incubation assays on PMN + *E. bovis* sporozoites in the presence of wortmannin (PI3K and autophagy inhibitor) and rapamycin (mTOR inhibitor, an activator of autophagy). Representative images are shown in **Figure 3A** (third and fourth rows, respectively). When the percentage of LC3B-positive cells for each experimental condition was determined, a significant increase (p = 0.01) of autophagy-positive cells was only observed in conditions of plain sporozoite supplementation (**Figure 3B**). Treatments with wortmannin partially decreased this activation but on a non-significant level. Unexpectedly, also rapamycin showed insignificant inhibitory effects on the proportion of LC3B-positive cells in sporozoite-exposed PMN. Moreover, these results were also analyzed in terms of punctuated and diffuse LC3B fluorescent signals. In this context, the punctuated pattern increased in the presence of *E. bovis* (p = 0.03; **Figure 3C**) but not the diffuse pattern (p = 0.29; **Figure 3D**). Interestingly, the diffuse pattern was now increased significantly in the presence of the mTOR activator rapamycin (p < 0.001; **Figure 3D**).

### *E. bovis* sporozoite-induced NET is dependent of the PMN:parasite ratio

**Figure 4:**
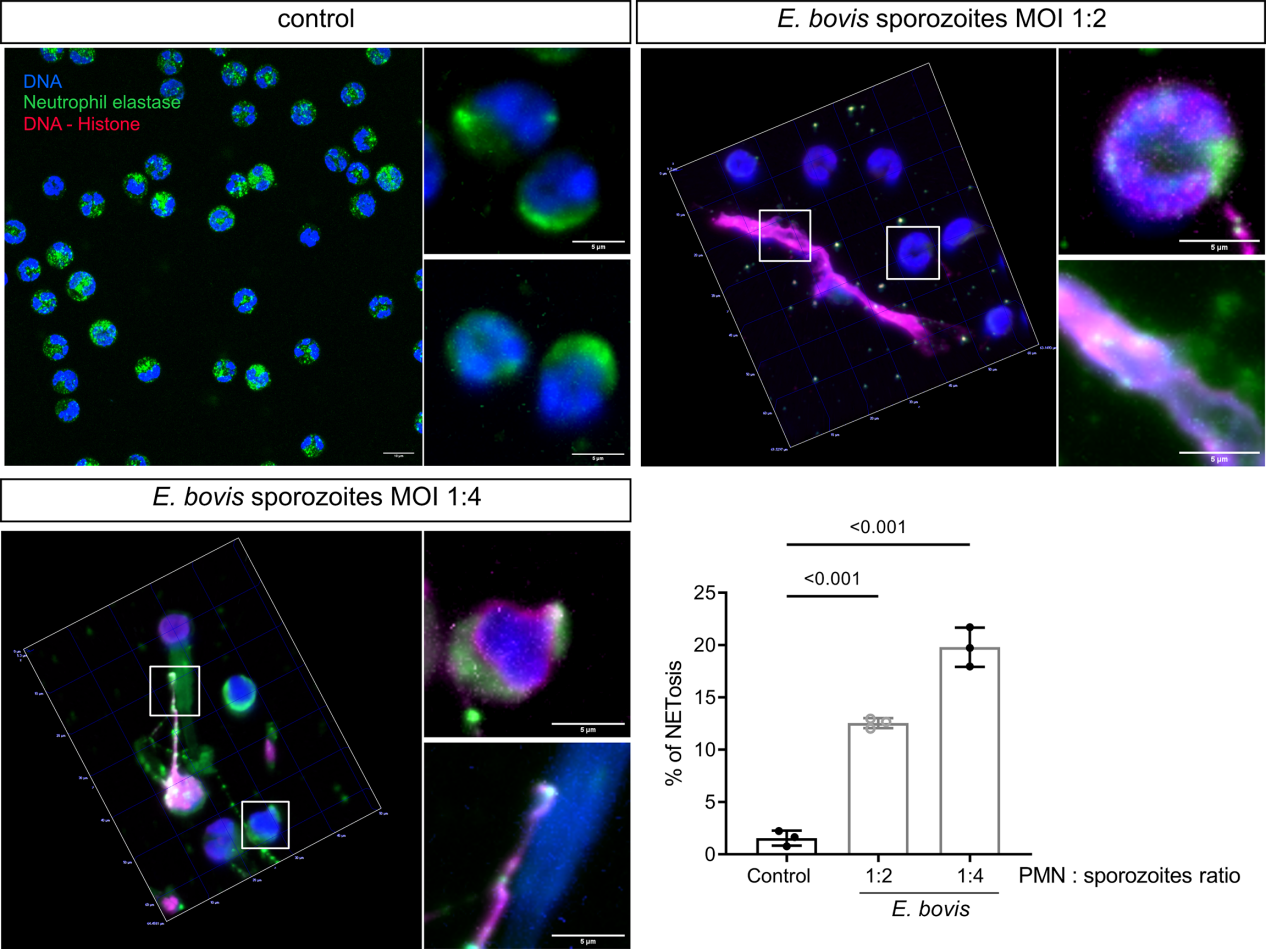
*E. bovis* sporozoite-induced NET formation is dependent on the PMN:parasite ratio. PMNs (2.5 × 10^5^) were seeded in poly-L-lysin-coated coverslips, and *E. bovis* sporozoites were added at 1:2 and 1:4 PMN:sporozoite ratios. After 120 min of co-incubation, the samples were fixed and then immunodetection of DNA–histone complex (red) and neutrophil elastase (NE; green) was performed. DNA was stained with DAPI (blue). Exemplary illustrations of unstimulated PMN PMN confronted with *E. bovis* sporozoites at 1:2 and 1:4 ratios. The percentage of cells forming NETs was quantified by a semiautomatic method and represented as mean ± SEM . p-values were calculated using a repeated measures (paired) ANOVA followed by a Dunnett’s multiple-comparison test analysis (n = 3) .

Activation of bovine PMN after confrontation with *E. bovis* sporozoites was evaluated further by determining the NET formation induced by *E. bovis* at PMN:sporozoite ratios of 1:2 and 1:4 after 2 h of co-incubation. The percentage of NET-forming PMN was determined according to Brinkmann et al. (51) using an anti-DNA–histone complex and anti-NE antibodies (**Figure 4**). Chromatin was counterstained by DAPI. NET formation was observed as chromatin structures being released from PMN and positive for NE and histone–DNA complex. Unstimulated PMN were used as negative controls (**Figure 4**). Current data showed that *E. bovis* sporozoites in a 1:2 PMN:parasite ratio induced NETs in 12.55 ± 0.48% of PMN versus 1.56 ± 0.72% of unstimulated PMN (**Figure 4**; p < 0.001). When a 1:4 PMN:parasite ratio was analyzed, 19.81 ± 1.87% of PMN were NET-positive indicating that *E. bovis*-induced NET formation is dependent on the PMN:*E. bovis* sporozoite ratio (**Figure 4**; p < 0.001).

### Vital NETosis occurred rapidly in bovine PMN exposed to motile *E. bovis* sporozoites

**Figure 5:**
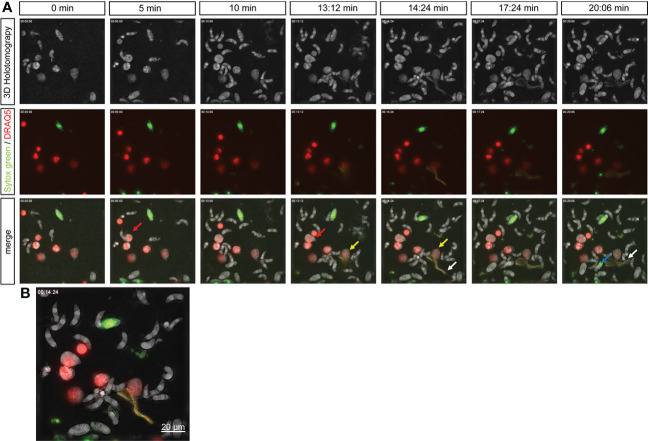
Live-cell imaging of vital NETosis performed by *E. bovis* -exposed PMN. Time lapse of a holotomographic video generated by 3D Cell Explorer (Nanolive) on the interaction between bovine PMN and live *E. bovis* sporozoites. The interactions between PMN and sporozoites were monitored for a total 100 min. The complete video of the interaction is available in the supplementary material. Panel **(A)** shows the 3D holotomography, green–red channels, and the merge. The nuclei of PMN were stained with DRAQ5 (red), extracellular DNA (as a marker of NETosis) with SYTOX green. Red arrow: PMN being invaded by a *E. bovis* sporozoite. Yellow arrow: PMN releasing NETs. White arrows: NET structure. Blue arrow: dead sporozoite. Numbers indicate the time points of interaction between PMN and *E. bovis* sporozoites. **(B)** shows a merge of all channels at 14 min of interaction and illustrates the formation of NETs and the contact of this DNA structure with a *E. bovis* sporozoite.

A holotomographic analysis with 3D Cell Explorer Fluo (Nanolive) on early interactions between bovine PMN and viable and motile *E. bovis* sporozoites was performed (please refer to **Supplementary video 1**). Activation of bovine PMN after addition of motile parasites to imaging media was evident by the observation by rapid pseudopod formation and increased kinetic activities. These changes in morphology and motility were documented for 100 min. **Figure 5** shows representative images of interactions between PMN and motile *E. bovis* sporozoites. The imaging media contained DRAQ5 (red) to stain PMN nuclei and SYTOX Orange to stain extracellular DNA, as a component of NETs. Here, six PMN nuclei were stained red and one dead *E. bovis* sporocyst was SYTOX Green-positive (**Figure 5A**) at time point 0. After 13 min of interaction, extrusion of a DNA diffuse web-like structure was observed in one of the analyzed PMN (white arrow, green staining in **Figure 5A** and zoomed in **Figure 5B**), which was in close contact to motile sporozoites. This spread NET (*spr*NET) became larger and was located in close proximity to sporozoites during the total incubation period. The PMN which released *spr*NET (yellow arrow) were alive during the whole process and died at minute 45 (i.e., 32 min after *spr*NET extrusion), thereby suggesting the cell process of vital NETosis. Of note, some sporozoites which were exposed to *spr*NET or in direct contact this NET structure died after 20 min (blue arrow).

Worthwhile to mention is that at minute 9 of coculture, one *E. bovis* sporozoite actively invaded a bovine PMN (please refer to Video 1, **Figure 5**, red arrow), and the whole invasion process took 36 s. The parasite moved inside the cytoplasm—as illustrated by the deformation of the invaded PMN cell—for less than a minute and even tried to escape. Then, a slow retrograde movement of the sporozoite back into the cell was seen, a process that took in total of 7.5 min. Thereafter, the invaded PMN began its nuclear expansion and finally died at minute 35, with the sporozoite still inside the cytoplasm (**Figure 5A** red arrow).

### Effects of extracellular pH on *E. bovis*-triggered NETosis

**Figure 6:**
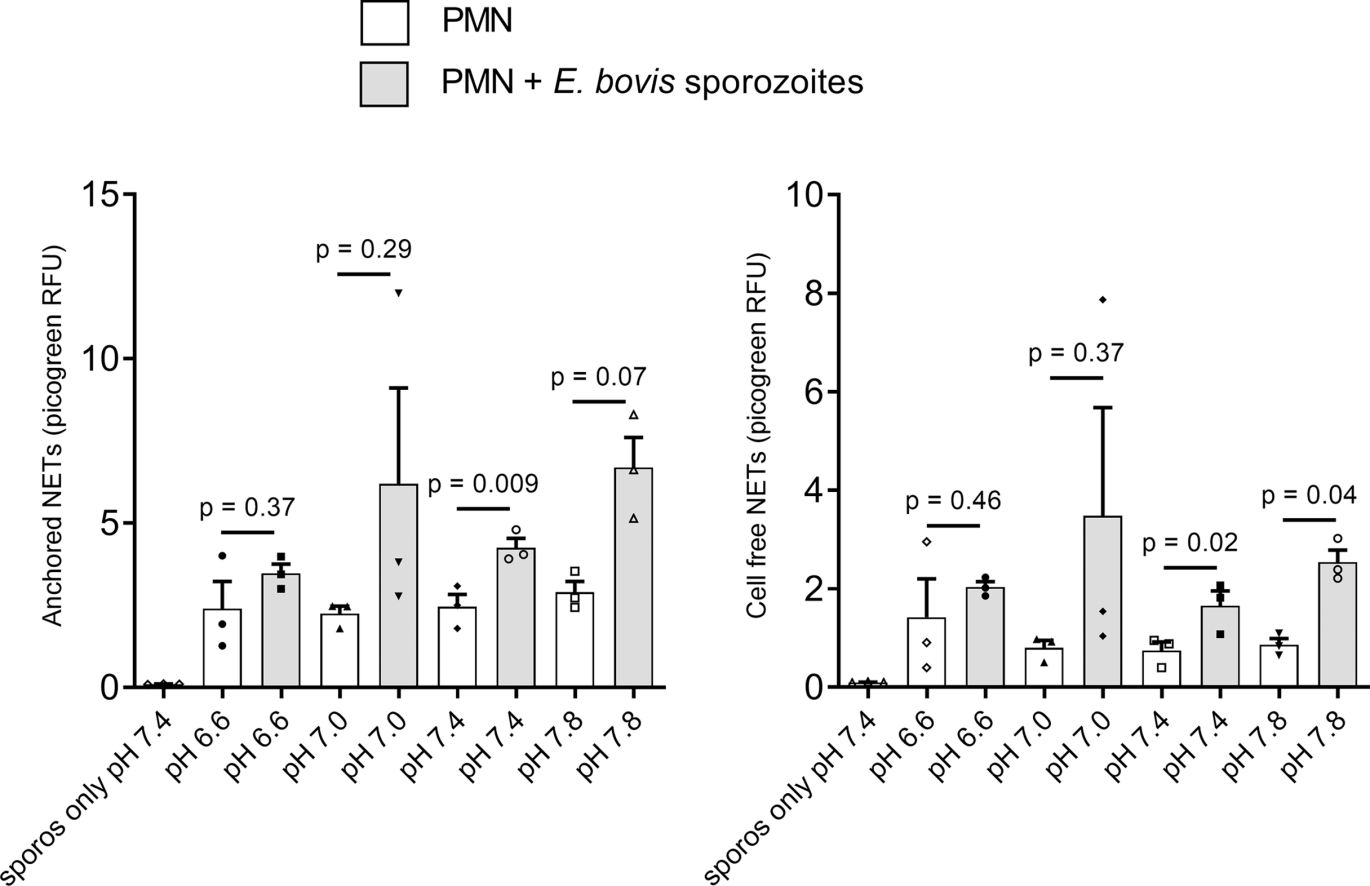
Effects of extracellular pH on *E. bovis* sporozoite-induced NETosis. Bovine PMN (n = 3) were suspended in RPMI 1640 media adjusted to different pH (6.6, 7.0, 7.4, and 7.8) levels and then exposed to *E. bovis* sporozoites. After 2 h of incubation, samples were centrifuged at 300 × g for 5 min. The supernatants were collected for cell-free NET measurement, and the pellets were used for anchored NET estimation. Extracellular DNA was detected and quantified by picogreen-derived fluorescence intensities using an automated multi-plate reader. Values are presented as mean ± SEM in the graphs. p values were calculated by a paired two-tailed t-test analysis, comparing the PMN alone vs. the *E. bovis*-confronted PMN at each pH (n = 3).

Since lactate or other proton-based efflux revealed crucial for NET formation and considering the observed ECAR-related effects in parasite-stimulated PMN, we here additionally studied the effects of different extracellular pH conditions (i.e., pH of 6.6, 7.0, 7.4, and 7.8) on sporozoite-triggered cell-free and ‘anchored’ NETosis phenotypes. Overall, only moderate effects were detected at different pH conditions, evidencing that the most consistent response is observed at pH 7.4 (p = 0.009 and p = 0.02 for anchored and cell-free NETs, respectively) (**Figure 6**). *E. bovis* sporozoite-induced NETosis depended on ATP synthase and lactate dehydrogenase activities.

We next investigated the relevance of single metabolic pathways during NETosis *via* the use of selected metabolic inhibitors interfering with distinct steps of glycolysis, glutaminolysis, and ATP generation. In detail, we studied sporozoite-induced NET formation and the related impact of PMN pretreatments with chemical compounds acting on the glucose lactate and citric acid cycle axis (FDG: blocks glycolysis at the hexokinase level; oxamate: inhibits lactate formation by blocking lactate dehydrogenase; oxythiamine: blocks pyruvate conversion to acetyl-CoA *via* pyruvate dehydrogenase inhibition, succinyl-CoA production by inhibition of α-ketoglutarate dehydrogenase as well as the non-oxidative pentose phosphate pathway by inhibiting transketolase; DCA: activates the conversion of pyruvate to acetyl-CoA by inhibiting pyruvate dehydrogenase kinase) and on mitochondrial ATP regeneration by inhibiting ATP synthase within the mitochondrial respiration chain (oligomycin). A scheme illustrating the inhibitors and their corresponding targets is shown in **Figure 8**.

**Figure 7:**
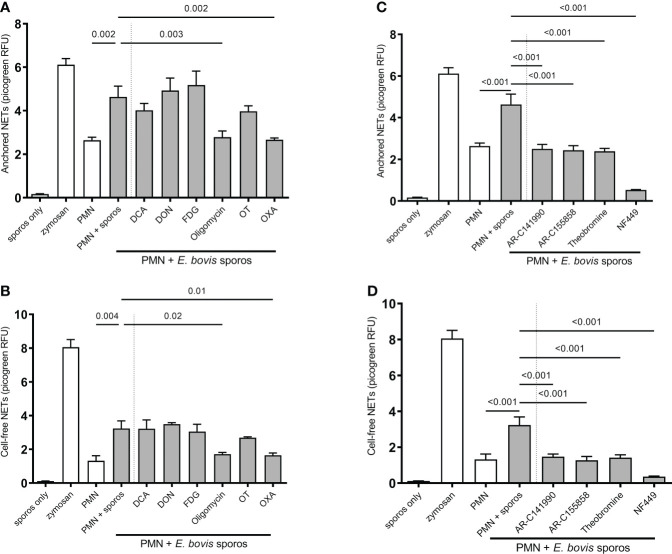
Effects of glycolysis, glutaminolysis, purinergic signaling (P2X1, P1A1), and monocarboxylate transporter (MCT) inhibition on *E. bovis* sporozoite-induced NETosis. Bovine PMN were pretreated for 30 min with FDG (2 mM), DCA (8 mM), OT (50 µM), OXA (50 mM), oligomycin (5 µM), DON (4 µM), NF449 (100 µM), theobromine (100 µM), AR-C141990 (1 µM), and AR-C155858 (1 µM), followed by the exposure to *E. bovis* sporozoites (ratio 1:4). For negative controls, PMN in plain, serum-free medium RPMI 1640 without phenol red was used. Stimulation of PMN with zymosan (1 mg/ml) served as a positive control. After 2 h of incubation, samples were directly centrifuged at 300 × g for 5 min. The pellets were used for ‘anchored’-NET estimation **(A, B)**, and the supernatants were collected for ‘cell-free’-NET measurements **(C, D)**. Extracellular DNA was detected and quantified by picogreen-derived fluorescence intensities using an automated multi-plate reader. All data were performed and analyzed by one-way ANOVA followed by Dunnett’s multiple-comparison test in GraphPad software to calculate the p-values. Data are presented as mean ± SEM in the graphs (n=3).

Quantification of ‘anchored’ and ‘cell-free’ NET phenotypes confirmed that sporozoites indeed triggered exposed bovine PMN to release both forms of NETs (**Figures 7A–D**). Functional inhibition experiments showed that this process seemed independent of glycolysis since FDG treatments failed to block NET formation (**Figures 7A, B**). Overall, the formation of both parasite-triggered ‘anchored’ and ‘cell-free’ NETs was significantly reduced by oxamate treatments (OXA; treated PMN + sporozoites vs. non-treated PMN + sporozoites, oxamate: ‘anchored’ *p* = 0.002, ‘cell-free’ *p* = 0.01) which indicated a key role of lactate generation during the NETosis process (**Figures 7A, B**). In addition, a significant decrease of ‘cell-free’ and ‘anchored’ NET formation was observed in the case of oligomycin A treatments (treated PMN + sporozoites vs. non-treated PMN + sporozoites, oligomycin A: ‘cell-free’ *p* = 0.002, ‘anchored’ *p* = 0.003), suggesting that efficient *E. bovis* sporozoite-induced NET formation was also dependent on ATP synthase activities. In contrast, treatments with DON, DCA, and oxythiamine failed to influence sporozoite-induced NETosis (**Figures 7A, B**).

**Figure 8:**
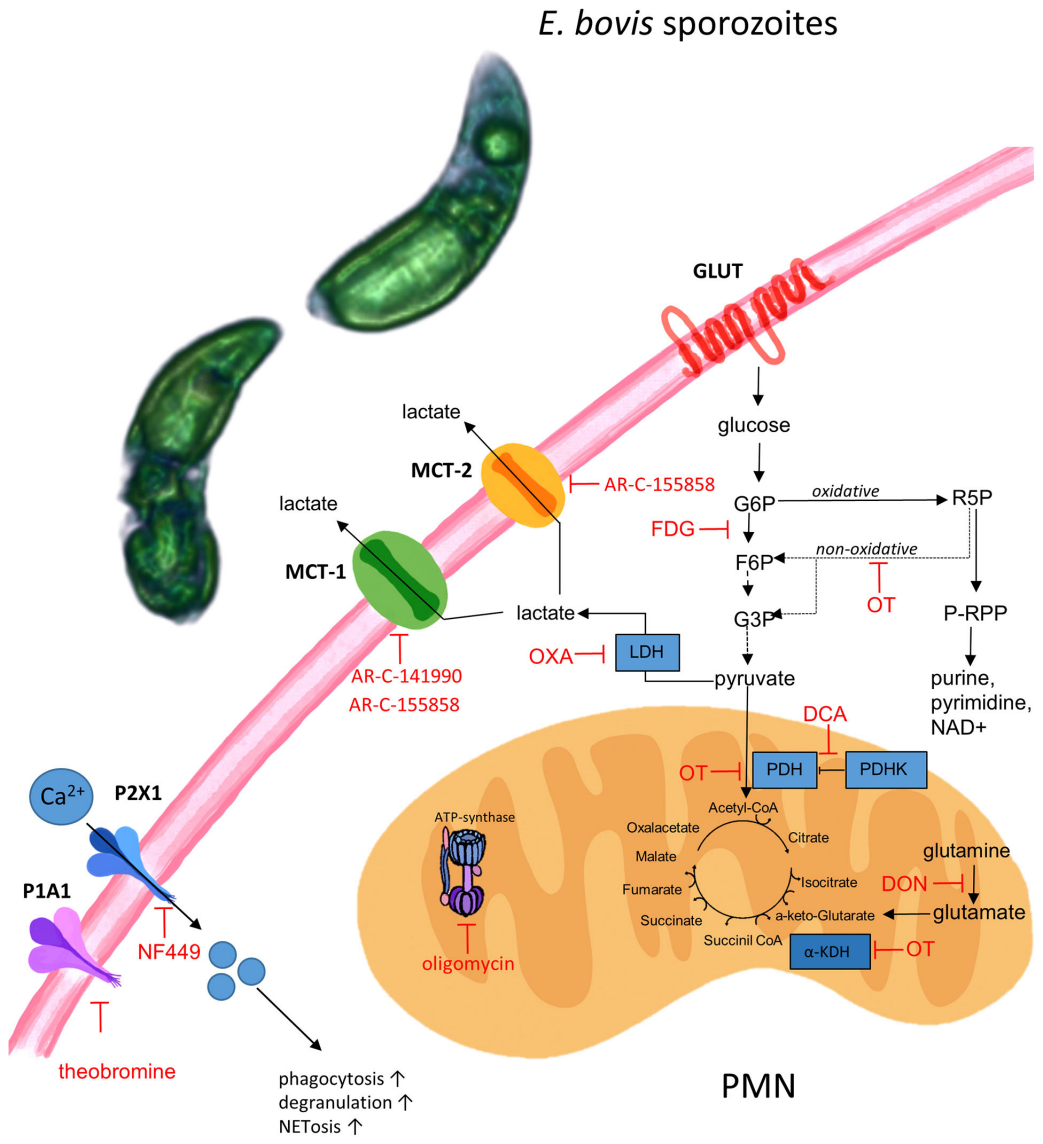
Schematic illustration of metabolic pathways and targets of inhibitors used in this study. FDG, fluoro-2-deoxy-D glucose; DCA, dichloroacetate; OT, oxythiamine; OXA, oxamate; DON, 6-diazo-5-oxo-L-norleucine, oligomycin, NF449 (inhibitor of P2X1 receptor), theobromine (inhibitor of P1A1 receptor), AR-C141990 (MCT1 inhibitor), and AR-C155858 (inhibitor of MCT1 and MCT2). *E. bovis* sporozoites can activate bovine PMN due to a direct interaction to the cell or due to *E. bovis*-secreted soluble mediators. Activation of *E. bovis* confronted PMN triggers purinergic signaling (P2X, P1A receptors), monocarboxylate transporter function, and metabolic switches in order to sustain effector mechanisms as ROS production, phagocytosis, and NET formation. Pharmacological targeting of some of the actors demonstrate the fundamental role of energy-related molecules in *E. bovis*-induced PMN response.

### *E. bovis* sporozoite-induced NETosis is dependent on monocarboxylate transporters and purinergic receptors

Given that lactate synthesis seemed of major importance during parasite-triggered NETosis, we additionally studied the relevance of lactate efflux in sporozoite-exposed PMN by applying chemical blockers of MCT which control the proton-linked transport of monocarboxylates, such as L-lactate, pyruvate, and ketone bodies, across the plasma membrane (30). Indeed, pretreatments of PMN with AR-C 141990 (blocks MCT1) and AR-C 155858 (blocks MCT1 and MCT2) both significantly reduced sporozoite-induced ‘cell-free’ and ‘anchored’ NET formation (treated PMN + sporozoites vs. non-treated PMN + sporozoites for both inhibitors: ‘anchored’ *p* ≤ 0.0001, ‘cell-free’ *p* ≤ 0.0001) (**Figure 7C, D**).

To further elucidate the relevance of purinergic signaling pathways in *E. bovis* sporozoite-induced NETosis, PMN were pretreated with theobromine (inhibits P1A1 receptor-mediated purinergic signaling) and NF449 (blocks P2X1 receptor-mediated purinergic signaling). As an interesting finding, we here showed that NF449 pretreatments entirely abolished *E. bovis* sporozoite-induced ‘cell-free’ and ‘anchored’ NET formation (levels were even below those of plain PMN) when compared to non-treated controls (treated PMN + sporozoites vs. non-treated PMN + sporozoites: both *p* < 0.001) (**Figures 7C, D**). In line, theobromine treatments also significantly reduced the formation of both types of NETs (treated PMN + sporozoites vs. non-treated PMN + sporozoites: ‘anchored’ *p* < 0.001 and ‘cell-free’ *p* ≤ 0.001) but to a lesser degree than NF449 (**Figures 7C, D**). These results strongly suggest that sporozoite-induced NETosis depends on both P2X1-mediated ATP binding and P1A1-mediated purinergic signaling.

## Discussion

In the current study, we investigated the relevance of selective metabolic pathways for *E. bovis*-induced NETosis. Overall, glycolysis is regarded as the main ATP source in mammalian PMN, thereby providing the energy required for different activities and PMN-derived effector mechanisms (59–62). In general, PMN are considered as highly glycolytic cells with minor energetic mitochondrial functions (63–65). To gain further insights into actual glycolytic responses of bovine PMN in reaction to parasite exposure, we here firstly analyzed OCR and ECAR of bovine PMN in the absence and presence of viable and motile *E. bovis* sporozoites. The Seahorse XF Analyzer directly measures two parameters, OCR which is extrapolated to mitochondrial respiration and ECAR which correlates with the glycolytic activity of the cells (48), based on the fact that the process of glycolysis results in acidification of the cell culture media which is here measured as proton efflux rate (PER) (25, 66, 67). Human PMN, activated by PMA, show OCR and ECAR increases, a phenomenon that was considered as indicative of PMN dependence on glycolysis during the oxidative burst and cellular activation (68). Indeed, human PMN challenged with *S. aureus* showed increased ECAR, indicating that glycolysis is also important in the PMN response against microorganisms (69). In line, we here detected a significant OCR increase in PMN upon sporozoite exposure, thereby indicating a parasite-driven activation of PMN-derived mitochondrial respiration (25, 43, 60). When we tested glycolytic responses under stress conditions, and in the absence of antimycin and rotenone, we found a significant increase in both glycolysis and glycolytic capacity in *E. bovis* sporozoite-exposed PMN. When glucose was injected, an increase in ECAR due to the formation of lactate was detected under both conditions, i.e., in the presence and absence of sporozoites, thereby mirroring enhanced glycolytic activity in PMN which is considered as the normal rate of glycolysis (25, 62). As an interesting finding, this glycolytic parameter was found to be significantly higher in parasite-exposed than in non-exposed bovine PMN. Furthermore, PMN were treated with oligomycin, which effectively blocks oxidative phosphorylation-driven ATP synthesis. To compensate ATP loss, PMN enhanced their glycolytic activity to a maximum level which resulted in a second step of ECAR upregulation, thereby reflecting the glycolytic reserve capacities of mammalian PMN (25). Again, this parameter showed to be significantly higher in parasite-exposed PMN. This finding in principle agreed with Mookerjee et al. (66), who found that the maximum glycolytic capacity of cells increases with enhanced current metabolic energy demand. Toward the end of glycolytic stress tests, glycolysis was inhibited by 2-DG, which permitted to measure low non-glycolytic ECAR levels thereby proving that previous reactions were indeed due to glycolytic responses. Overall, the current data confirmed the importance of glycolysis in apicomplexan-triggered activation of PMN and a high neutrophilic glycolytic capacity in moments of cellular stress (26, 70).

Autophagy is an intracellular programmed degradation system, which recycles unnecessary or damaged components including proteins and organelles, and is essential in cellular response to different stress factors such as hypoxia, inflammation, and oxidative burst (12). Recently, autophagy was correlated with a simultaneous NET formation in bovine PMN exposed to the closely related apicomplexan parasite *B. besnoiti* (13), and we evaluated autophagy in *E. bovis* sporozoite-exposed PMN. Here autophagosome formation was visualized by LC3B-based immunostaining. Of note, phagocytosis of *E. bovis* by bovine PMN is not necessary for the formation of LC3B puncta, indicating the possibility that *E. bovis*-derived soluble molecules induces autophagy in bovine PMN. Autophagosomes are double-membraned vesicles formed during autophagy, which represent characteristic markers of autophagy. Confocal microscopy showed that confrontation of PMN with sporozoites caused a significant increase in autophagosome formation. Our results agreed with those obtained by Zhou et al. in bovine PMN confronted to tachyzoites of *B. besnoiti* (13). However, given that neither rapamycin nor wortmannin treatments significantly altered sporozoite-driven NETosis, it is contradictory with other studies showing that the mTOR pathway indeed played a role in NET formation *via* regulation of autophagic pathways (49).

In line with observations for other apicomplexan parasites, such as *B. besnoiti* (58) and *N. caninum* (71), the *E. bovis-*induced NET formation was revealed as dependent on the PMN:parasite ratio, confirming earlier observations on *E. bovis*-induced NETs (7). These data were consistently obtained after 120 min of co-incubation, which is in agreement with the previous report on *E. bovis*-confronted bovine PMN (7). As expected, not all sporozoite-exposed PMN released NETs. As such, *E. bovis-*induced NETs were detected in 19.81% of bovine PMN. This proportion matches with that reported for *B. besnoiti* (15%) using the same PMN:parasite ratio (72). Regarding PMN receptors involved in this process, it was recently described that TLR-2 and TLR-4 are involved in *E. bovis*-induced bovine NETosis (73). This adds novel data on pathogen recognition receptors (PRRs) since the previous data exclusively indicated CD11b as also partially involved in *E. bovis*-induced NET formation (6). Whether these receptors are also involved in NETosis induced by other apicomplexan parasites remains unknown and is matter of current ongoing research.

So far, the knowledge in early innate immune reactions against cattle *Eimeria* spp. and other ruminant species is incomplete. The first contact between parasite and the host innate immune system is considered to be decisive for the outcome of eimeriosis (74). NETs being formed by bovine PMN in response to *E. bovis* sporozoites and other ruminant eimerian species such as caprine *Eimeria ninakohlyakimovae* and *Eimeria arloingi* have been reported previously (75, 76), but, so far, no data were available on vital NETosis. Thanks to 3D live-cell imaging, we were able to document for the first time vital NETosis in PMN stimulated with highly motile *E. bovis* sporozoites. This novel tool allowed us to capture in real time the release of a DNA-rich structure, similar to the *spr*NET phenotype, from an activated PMN after a short time of sporozoite exposure while the PMN remained vital. Further research should focus on the proportion of cells performing vital NETosis and on the coexistence of suicidal and vital NETosis during parasitic stimulation. In addition, it would be of high interest to elucidate the molecular stimuli necessary for PMN to choose either pathway leading to rapid or slow entrapment.

As an interesting finding, *E. bovis* sporozoite invasion of a PMN was also documented here. Likewise, invasion of leukocytes by *E. bovis* was previously reported for monocytes and PMN (74). Nevertheless, *E. bovis* PMN invasion was reported as a rare event, since sporozoites failed to develop further in professional phagocytes, e.g., in a permanent bovine macrophage cell line (BOMAC) and primary bovine macrophages (74).

Inhibition of glycolysis by 2-FDG treatments failed to reduce parasite-triggered DNA release. Considering the increase of glycolytic responses upon parasite exposure describe above, these reactions must rather be attributed to the activation process itself and/or other effector functions, such as chemotaxis, chemokine/cytokine synthesis, degranulation, or even phagocytosis, than NETosis. In line with this, glycolytic requirements for PMN-derived activation-related processes, such as chemotaxis or phagocytosis, were already reported (59, 60, 77–79). In agreement with the current data, exposure of PMN to *B. besnoiti* tachyzoites also led to enhanced glycolytic responses (as measured by upregulated glucose consumption), but parasite-triggered NETosis was not influenced by FDG treatments (29). In contrast, Rodríguez‐Espinosa et al. (26) showed that glycolysis inhibition caused a reduction of PMA-mediated human NETosis. This discrepancy in NET-related findings may rely on differences in the model (human vs. bovine PMN) or discrepancies of NET stimulators (parasites vs. PMA) [11].

According to the current findings, ATP synthesis is of major importance for efficient *E. bovis* sporozoite-triggered NET formation. ATP is produced either by glycolysis or by mitochondrial respiration. Interestingly, PMN contains only a few mitochondria (63, 65) and previous research suggested that these organelles do not play a key role in PMN-related energy metabolism (70). However, this point of view has been challenged recently (36, 80). Nevertheless, while blockage of glycolysis failed to impair NETosis, blockage of mitochondrial ATP synthesis by oligomycin significantly reduced sporozoite-triggered NET formation. These findings are consistent with previous research on PMA-induced NETosis (26) and on oligomycin-induced blockage of *B. besnoiti* tachyzoite-mediated NET formation (29). Consequently, mitochondrial ATP production seems of general relevance for proper NETosis function. Notably, oligomycin treatments also impaired respiratory burst activity in human and bovine PMN (63, 81), which is mechanistically linked to the dynamic NETotic process. Interestingly, a recent study reported that mitochondrial ATP was indeed required for human PMN activation and that inhibition of mitochondrial ATP synthesis had only minor effects on intracellular ATP levels but inhibited the release of ATP into the extracellular space in human PMN (80). Importantly, extracellular ATP acts as a pivotal messenger molecule promoting cell-to-cell communication and driving purinergic signaling-dependent mechanisms *via* various purinergic receptors. As such, it was demonstrated that extracellular ATP hydrolysis inhibited PMN migration and that inhibition of purinergic signaling blocked PMN activation and impaired innate host responses to bacterial infection (82). Therefore, we here also studied the relevance of purinergic signaling. Overall, purinergic receptors are involved in PMN chemotaxis, phagocytosis, oxidative burst, apoptosis, and degranulation (82–85). It has been reported that extracellular ATP regulates PMN chemotaxis *via* P2Y2 receptors (86) and that P2Y receptors are involved in PMN adhesion to the endothelium (87, 88). Interestingly, PMN-derived P2X and P2X7R surface receptors are required for NLRP3-mediated inflammasome activation and bacterial killing (89). We here found that the P2X1 receptor plays a crucial role in *E. bovis* sporozoite-induced NETosis since its inhibition *via* NF449 treatments entirely blocked parasite-triggered NET formation. This finding is supported by recent data on the role of P2-receptors as important players in NET formation induced by *Neospora caninum* (42), *B. besnoiti* (29), and *T. brucei brucei* (43) as well as in aggregated NET (*agg*NET) formation induced by gout-associated monosodium urate crystals (90). Besides the pivotal role of the P2X1 receptor, we here additionally identified P1A1-mediated purinergic signaling as a key event in *E. bovis* sporozoite-mediated NETosis since theobromine treatments also led to a significant reduction of this effector mechanism in bovine PMN.

Inhibitors of the lactate pathway confirmed the importance of this metabolite in NETosis. Lactate is not exclusively produced *via* the glucose–pyruvate–lactate axis but may also be synthesized by other metabolic pathways, such as pentose phosphate and Krebs cycle (91). In addition, lactate signals to formation of NETs, *via* the NOX/ROS axis (34) and citrullination of histones *via* PAD4 (31). In the current study, treatments with oxamate, AR-C 141990, and AR-C 155858 all efficiently blocked *E. bovis*-induced formation of ‘cell-free’ and ‘anchored’ NET phenotypes. Oxamate inhibits lactate dehydrogenase, thereby reducing lactate release and regeneration of NAD^+^ (92) which both may influence NETosis. Our results are in accordance with a previous report showing that oxamate inhibits PMA and A23187-induced NETosis (34). Since *E. bovis*-triggered NETosis was highly dependent on MCT activities, we here also studied the effect of extracellular pH in *E. bovis* sporozoite-mediated NETosis. It was described that extracellular pH modulates the functions of immune cells (93), including PMN (94). Thus, extracellular acidification caused delayed apoptosis, enhanced endocytosis, and inhibited bacterial killing in human PMN (95). Likewise, intracellular killing mechanisms and ROS-dependent NET formation were also inhibited by extracellular acidification in bovine PMN (27). The current data indicated that extracellular alkalization led to increased *E. bovis* sporozoite-induced NETosis, which is in line with NET-related reports in the human system (52, 96). This finding may be based on alkalinization-induced cytosolic calcium fluxes (97), which are required for PAD4-mediated histone citrullination and subsequent NETosis (42). Consequently, acidification commonly found in conditions of inflammation may impair proper NETosis in the *in vivo* situation.

In summary, this study provides a better understanding of relevant metabolic pathways, purinergic signaling, pH conditions, and PMN activation during *E. bovis* sporozoite-induced NETosis. Additionally, the current data give first evidence on *E. bovis* sporozoite-induced vital NETosis and the formation of autophagosomes.

## Data availability statement

The original contributions presented in the study are included in the article/**Supplementary Material**. Further inquiries can be directed to the corresponding author.

## Ethics statement

The animal study was reviewed and approved by Ethics Commission for Experimental Animal Studies of Federal State of Hesse (Regierungspräsidium Giessen; A2/2016; JLU-No. 589_AZ and G16/2017, JLU-No. 835_GP).

## Author contributions

CH, AT, and IC: conceptualization, designed the project and experiments. EZ carried out the exDNA and LC3B experiments. SL-O: carried out the Seahorse and Nanolive experiments, performed the excystation of *Eimeria bovis*, and analyzed the data. IC and ZV: prepared and analyzed the data. SL-O and IC: prepared the original draft. SL-O, ZV, and IC: prepared the figures. ZV and MR: performed immunofluorescence, generated confocal images, and determined the percentage of NET formation. RB and PA: contributed to the MCT and purinergic receptor inhibitor experiment design and reagents. JC-G: conceptualization and critical review of the manuscript. All authors reviewed the manuscript. All authors contributed to the article and approved the submitted version.

## Funding

The present work was financed by the “Deutsche Forschungsgemeinschaft” (DFG project: TA291/4-2). SL-O was funded by a DAAD Doctoral Fellowship (Stipendium 57381410, 2018/19). MR was funded by the Erasmus+ mobility program and the pre-doctoral formation program of Research personnel in Canarias. "Conserjería de Economía, Conocimiento y Empleo"; co-funded by the European Social Fund (TESIS2021010015).

## Acknowledgments

The authors would like to acknowledge all staff members of the Institute for Parasitology, JLU Giessen, Germany. We also would like to acknowledge Ulrich Gärtner, Institute of Anatomy and Cell Biology, JLU Giessen, Germany, for giving us access to the confocal microscopy unit. We further thank all staff members of JLU Giessen large animal teaching and research station Oberer Hardthof. CIBAV of the School of Veterinary Medicine express their gratitude to the Strategy of Consolidation of Research Groups CODI 2018-2019 of the University of Antioquia, Medellin, Colombia. Finally, we would like to acknowledge Oliver Bender of the Ubl butchery in Langsdorf, Germany, for his constant and kind supply of bovine bile.

## Conflict of interest

The authors declare that the research was conducted in the absence of any commercial or financial relationships that could be construed as a potential conflict of interest.

## Publisher’s note

All claims expressed in this article are solely those of the authors and do not necessarily represent those of their affiliated organizations, or those of the publisher, the editors and the reviewers. Any product that may be evaluated in this article, or claim that may be made by its manufacturer, is not guaranteed or endorsed by the publisher.

## Supplementary material

Supplementary Video 1 (*E. bovis* triggers NETs observed by live cell 3D microscopy).
