# Supplementary material for: Glycolysis, monocarboxylate transport, and purinergic signaling are key events in Eimeria bovis-induced NETosis
Source: Front Immunol. 2022 Aug 11;13:842482. doi: 10.3389/fimmu.2022.842482 (PMC9403323; doi:10.3389/fimmu.2022.842482)
Supplement: Supplementary file 1 [file DataSheet_1.pdf]

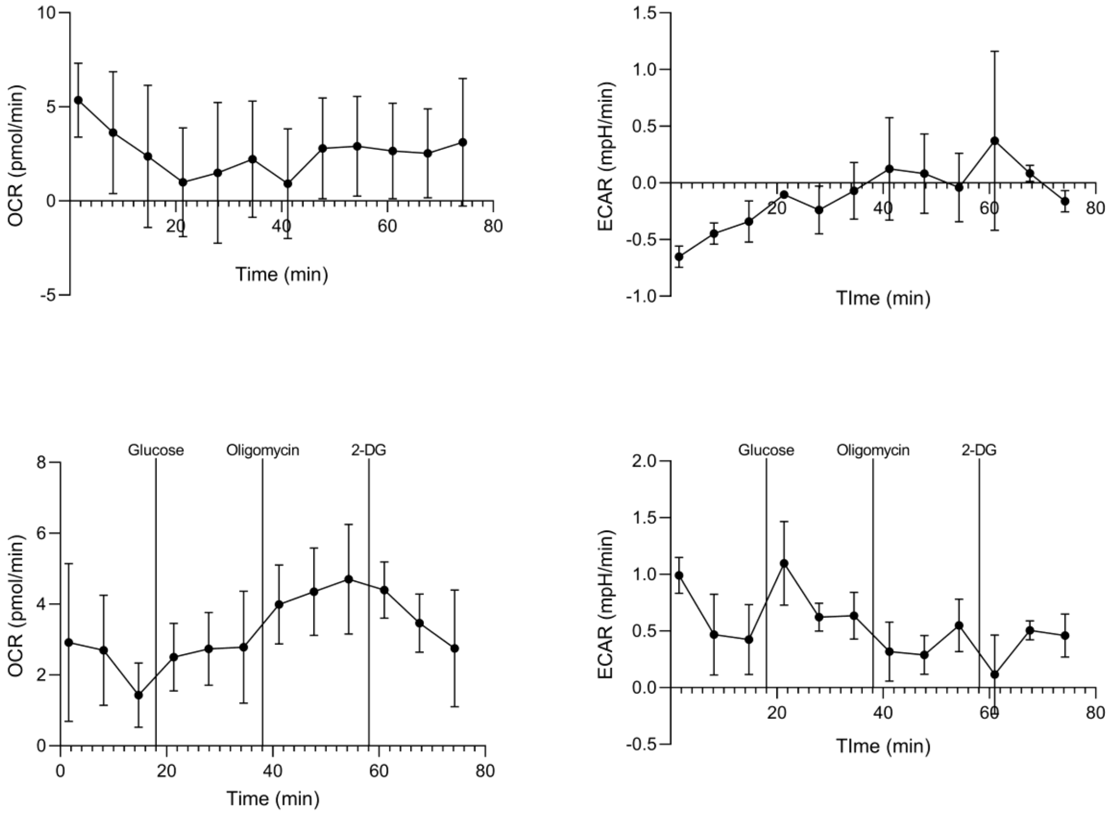

Metabolic activity of *E. bovis* sporozoites analysed by Seahorse technology. 200.000 freshly excystated *E. bovis* sporozoites were seeded in a 8-well XF seahorse plate and the basal metabolic activity or the response to sequential injections of chemicals from the glycolytic rate assay kit was evaluated in the same conditions that were used to obtain the data of figures 1 and 2
